# The effect of C–C motif chemokine ligand 2 supplementation on *in vitro* maturation of porcine cumulus-oocyte complexes and subsequent developmental competence after parthenogenetic activation

**DOI:** 10.3389/fvets.2023.1136705

**Published:** 2023-03-13

**Authors:** Sohee Kim, Dongjin Oh, Hyerin Choi, Mirae Kim, Lian Cai, Ali Jawad, Zheng Haomiao, Joohyeong Lee, Eunhye Kim, Sang-Hwan Hyun

**Affiliations:** ^1^Laboratory of Veterinary Embryology and Biotechnology, Veterinary Medical Center and College of Veterinary Medicine, Chungbuk National University, Cheongju, Republic of Korea; ^2^Institute of Stem Cell and Regenerative Medicine, Chungbuk National University, Cheongju, Republic of Korea; ^3^Graduate School of Veterinary Biosecurity and Protection, Chungbuk National University, Cheongju, Republic of Korea; ^4^Laboratory of Molecular Diagnostics and Cell Biology, College of Veterinary Medicine, Gyeongsang National University, Jinju, Republic of Korea

**Keywords:** C-C motif chemokine ligand 2 (CCL2), porcine follicular fluid (pFF), *in vitro* maturation (IVM), embryonic development, parthenogenetic activation (PA)

## Abstract

Porcine embryos are used for a variety of applications. However, the maturation rate *in vitro* remains low, and novel *in vitro* maturation (IVM) techniques that facilitate the collection of mature oocytes are necessary. C-C motif chemokine ligand 2 (CCL2) is a key periovulatory chemokine present in cumulus-oocyte complexes (COCs). We aimed to examine the effects of CCL2 supplementation during IVM on oocyte maturation and embryonic development. The CCL2 concentration was significantly higher in porcine follicular fluid (pFF) derived from follicles >8 mm in size than in pFF derived from smaller follicles. There was a significant increase in *CCL2* mRNA levels in all follicular cells after IVM compared with that before IVM. We analyzed the localization of CCL2 and its receptor, the CCL2 receptor, in follicular cells. During IVM, different concentrations of CCL2 were added to COCs cultured in a maturation medium. After IVM, the group treated with 100 ng/mL CCL2 showed significantly higher metaphase II rates than the control group. All CCL2-treatment groups showed a significant increase in intracellular glutathione levels and a significant decrease in reactive oxygen species levels, compared to the control. In CCs treated with 100 ng/mL CCL2, the mRNA levels of *BAX, CASP3*, and *NPR2* were significantly decreased. Furthermore, the mRNA levels of *SOD1, SOD2*, and *CD44* were significantly increased. In oocytes treated with 10 ng/mL CCL2, mRNA levels of *BAX* and *CASP3* were significantly decreased, whereas, *NRF2* and *NPM2* were significantly increased. *ERK1* exhibited significantly increased mRNA expression in both CCs and oocytes treated with 10 ng/mL CCL2. The protein expression ratio of phosphorylated ERK1/2 to total ERK1/2 was significantly increased in CCs treated with 10 ng/mL CCL2. After parthenogenetic activation, cleavage rates were significantly improved in the 100 ng/mL CCL2 treatment group, and blastocyst formation rates were significantly enhanced in the 10 ng/mL CCL2 treatment group. Overall, our results suggest that IVM medium along with CCL2 improves porcine oocyte maturation and the development of parthenogenetically-activated embryos.

## 1. Introduction

Pigs and humans have similar anatomical structures, physiological features, and pathophysiological responses (1, 2). Therefore, they serve as good animal models for xenotransplantation and human disease research (3, 4). Porcine embryos are used for *in vitro* fertilization, intra-cytoplasmic sperm injection, sperm-mediated gene transfer, embryo cryopreservation, and the establishment of embryonic stem cells from early embryos for therapeutic research (5). Therefore, it is important to develop *in vitro* maturation (IVM) techniques that facilitate the collection of mature oocytes. To produce high-quality mature oocytes, nuclear and cytoplasmic maturation must occur simultaneously. Because this does not occur simultaneously *in vitro*, the maturation rate remains lower than in *in vivo* conditions, resulting in reduced embryonic development (6–8). The embryonic quality and developmental competence have been improved by the addition of supplements, such as growth factors, proteins, and antioxidants, to the culture medium used for porcine embryonic development (9).

Cytokines are essential regulators of folliculogenesis, ovulation, and corpus luteum formation. Their functions within the follicle include regulating cell proliferation and differentiation, follicle survival and closure, as well as oocyte maturation (10–12). For example, interleukins produced by various ovarian cell types participate in cell-to-cell communication in specific ovarian processes to regulate follicular development and atresia, ovulation, and steroidogenesis (13). The transforming growth factor-beta superfamily and its downstream effectors -Smad signaling, expressed at different stages of folliculogenesis, are multifunctional regulators of cell proliferation, differentiation, migration, and apoptosis. They also facilitate extracellular matrix formation (14, 15).

Chemotactic cytokines, known as chemokines, play critical physiological roles in ovarian function and ovulation (16). Several types of chemokine receptors are expressed in granulosa cells (GCs) and theca cells, which suggests that chemokines are involved in autocrine/paracrine signaling (17, 18). Paracrine and autocrine signals are crucial for the survival and development of the follicles. During the early stage of ovarian follicular development *in vitro*, paracrine signals, such as soluble cytokines, promote their survival (19, 20). The paracrine signaling pathway modulates the extracellular matrix of the cumulus cell (CC) (21). The C-C motif chemokine ligand 2 (CCL2), also known as monocyte chemoattractant protein-1 (MCP-1), recruits monocytes and macrophages and regulates their migration and infiltration (22). During follicle growth, macrophages localized in the theca cell layer increase in number and secrete growth factors and/or cytokines that, promote cell proliferation (23). CCL2 is produced by a variety of cell types. Its main receptor is the CCL2 receptor (CCR2) (24). During antral follicle growth, the levels of CCL2 originating from GCs and theca cells increase significantly (10, 25). In porcine ovaries, CCL2 prevents CC apoptosis *via* bone morphogenetic protein 15 (BMP15) and fibrillin-1 (FBN-1) (26). CCL2 modulates inflammation and endoplasmic reticulum stress, enhancing the proliferation of bovine endometrial epithelial cells (27). In feline cumulus-oocyte complexes (COCs), CCL2 increases the mRNA levels of important genes that are involved in ovulation *via* the CCR2 (28).

Previous studies have indicated that CCL2 levels are increased during follicular development and that CCL2 is associated with ovulation in COCs. Therefore, we hypothesized that CCL2 supplementation during porcine IVM would have a positive effect on oocyte development. In this study, we measured the concentration of CCL2 in porcine follicular fluid (pFF) using enzyme-linked immunosorbent assay (ELISA) and analyzed the expression level of *CCL2* mRNA in follicular cells before and after IVM. We determined the localization of CCL2 and CCR2 in follicular cells before and after IVM by immunostaining and evaluated the nuclear and cytoplasmic maturation after supplementing the IVM medium with CCL2. After IVM, the expression patterns of various genes in oocytes and CCs were determined. Finally, the embryonic developmental potential was evaluated after parthenogenetic activation (PA).

## 2. Materials and methods

### 2.1. Chemicals and reagents

Recombinant human CCL2/JE/MCP-1 chemokines were purchased from R&D Systems (Minneapolis, MN, USA). Unless otherwise stated, all chemicals and reagents used in this study were purchased from Sigma-Aldrich (St. Louis, MO, USA).

### 2.2. Measurement of CCL2 concentration in pFF

Ovary pairs from prepubertal three-way cross pigs (mixed Yorkshire, Landrace, and Duroc breeds) obtained from a local slaughterhouse, were collected for follicular fluid analysis. Aspirated porcine follicles were divided into three groups: small (1–2 mm), medium (3–7 mm), and large (≥8 mm) (29, 30). To eliminate debris and blood, the pFF was centrifuged for 20 min at 94 g and 4°C. The supernatant was extracted using a 10 cc disposable syringe (Sofjec; Hwajin Medical, Chungnam, Korea) with a 0.2 μm syringe filter (Corning, NY, USA) and frozen at −80°C. A porcine CCL2/MCP-1 ELISA kit (ES2RB; Invitrogen, Carlsbad, CA, USA) was used to measure the CCL2 concentration in the pFF samples, according to the manufacturer's instructions. The CCL2 concentration was determined using the mean value of four ELISA experiments, performed in duplicates.

### 2.3. Oocyte collection and IVM

Porcine ovaries were collected from local slaughterhouses and transported within 60 min to a laboratory where they were stored at 37°C in 0.9% NaCl. Oocyte collection and IVM were conducted as previously reported (31). A 10 cc disposable syringe with an 18-gauge needle was used to aspirate the follicular fluid from antral follicles (3–6 mm in size). The fluid was collected in a 15 mL conical tube, and debris was allowed to settle for 5 min, while the temperature was maintained at 37°C. After the supernatant was removed, precipitates were washed twice in HEPES-buffered Tyrode's medium (TLH) containing 0.05% (w/v) polyvinyl alcohol (PVA). Using a stereomicroscope, only COCs from the follicular fluid containing three or more uniform layers of compact CCs and homogeneous ooplasm were chosen. Approximately 60 COCs were placed in each well of a 4-well dish (Nunc, Roskilde, Denmark). Each well contained 500 μL of maturation medium (TCM199; Invitrogen) comprising 0.1% (w/v) PVA, 0.6 mM cysteine, 0.91 mM sodium pyruvate, 10 ng/mL epidermal growth factor, 75 μg/mL kanamycin, 1 μg/mL insulin, 10 IU/mL equine chronic gonadotropin (eCG), and 10 IU/mL human chorionic gonadotropin (hCG) (Intervet, Boxmeer, Netherlands). PVA was replaced with purified 10% (v/v) pFF in a general maturation medium for IVM. The selected COCs were cultured in a humidified chamber at 39°C with 5% CO_2_ and 95% air. COC cultures were maintained for 40–42 h. During the first 22 h, the COCs were maintained in a medium containing eCG and hCG, and during the subsequent 18–20 h, they were maintained in a hormone-free medium. Throughout the maturation period, CCL2 was added to each well at four different concentrations [0 (Control), 1, 10, and 100 ng/mL], determined as previously described (28).

### 2.4. Reverse transcription polymerase chain reaction

We used RT-PCR to examine *CCL2* expression in the three types of porcine follicular cells (oocytes, CCs, and GCs), before and after IVM. In general, IVM targets COCs, but we separately cultured it to confirm the mRNA levels of *CCL2* in GCs according to the presence or absence of hormone treatment. First, we isolated mural GCs and immature COCs from pFF samples precipitate collected as described in 2.3. The mural GCs were classified under a microscope except for COCs (32). Here, we decided to refer to these mural GCs after being immediately isolated from pFF as 'GCs before IVM'. Then, oocytes and CCs were separated from immature COCs using 0.1% (w/v) hyaluronidase and sampled for experiments. After 40–42 h of IVM, mature CCs and mature oocytes in the metaphase II (MII) stage were obtained. GCs were cultured separately but under the same conditions that were used for IVM. Referring to the previously described method for *in vitro* culture of porcine GCs, GCs were cultured in medium containing eCG and hCG for the first 22 h, followed by culture in hormone-free medium for 18–20 h (33). After 40–42 h of culture, we decided to refer to these as “GCs after IVM”.

RNA was extracted using TRIzol reagent (TaKaRa Bio, Otsu, Shiga, Japan). Complementary DNA (cDNA) was synthesized using a reverse transcription master mix (Elpis Bio, Daejeon, Korea), in accordance with the manufacturer's instructions. RT-PCR was conducted using 1 μL cDNA template, 10 pmol CCL2 forward and reverse primers (Macrogen, Seoul, Korea), two units of Taq polymerase (Elpis Bio), 2 μL 10X PCR buffer (Elpis Bio), and 5 pmol deoxyribonucleoside triphosphate mix (iNtRON Biotechnology, Seongnam, Korea). For RT-PCR amplification of oocytes, the following cycling conditions were used: 33 or 36 cycles of denaturation at 95°C for 30 s, annealing at 59°C for 30 s, and extension at 72°C for 30 s. Supplementary Table 1 lists all primer sequences. The mRNA expression values were standardized to that of 18S ribosomal RNA (*RN18S*). The RT-PCR products were analyzed by electrophoresis at 100 V for 25 min on a 1.25% agarose gel stained with RedSafe nucleic acid staining solution (iNtRON Biotechnology). A Bio-1000F gel imager (Microtek International, Hsinchu, Taiwan) was used to capture images of the gel.

### 2.5. Immunofluorescence staining

GCs, CCs, and oocytes before and after IVM were cultured as described in Section 2.4. COCs collected before and after IVM were separated from each other by physical pipetting using 0.1% (w/v) hyaluronidase. Immunostaining was performed as previously described (34). First, CCs and GCs before and after IVM were fixed in 4% paraformaldehyde in PBS for 10 min at 25°C (room temperature; RT). Oocytes before IVM and mature oocytes at the M II stage after IVM were fixed in 0.1% paraformaldehyde in PBS. Following fixation, all cells were washed thrice for 5 min with Dulbecco's phosphate-buffered saline (DPBS) containing CaCl_2_, MgCl_2_, and 0.1% (w/v) PVA and then permeabilized for 30 min with 0.5% Triton X-100. All cells were treated with Image-iT™ FX Signal Enhancer (Invitrogen, Carlsbad, CA, USA) for 30 min at RT to remove the non-specific background and then blocked for 30 min at RT with blocking buffer (Cell Signaling Technology, Beverly, MA, USA). After they were processed with the blocking buffer, the cells were incubated overnight at 4°C with the following primary antibodies: mouse anti-MCP-1 (cs-32771, 1:50 dilution in blocking buffer; Santa Cruz Biotechnology, CA, USA) and anti-CCR2 (NBP1-48338, Novus Biologicals, Littleton, CO, USA). The next day, all cells were washed thrice for 5 min with 0.1% PVA-PBS and then incubated for 1 h on a shaker with the following secondary antibodies: goat anti-mouse IgG (H + L) Alexa Fluor™ 488 (A11029, 1:200; Invitrogen, Carlsbad, CA, USA) and donkey anti-rabbit IgG (H + L) Alexa Fluor™ 594 (A21207, 1:200; Invitrogen). After removal of the secondary antibodies, all cells were washed thrice for 5 min with 0.1% PVA-PBS. The nuclei of all cells were counterstained with 10 μg/mL Hoechst-33342 for 10 min and mounted on glass slides using an anti-fade mounting medium (Molecular Probes, Eugene, OR, USA). A confocal laser microscope (Carl Zeiss, Thornwood, NY, USA) was used to examine the oocytes, and ZEN (blue edition) software was used to analyze all captured images. In negative controls, a blocking buffer made in the incubation medium was used instead of the primary antibody.

### 2.6. Assessment of nuclear maturation

The rate of nuclear maturation was evaluated after 40–42 h of IVM with CCL2 supplementation. CCs were removed from the oocytes by gently pipetting 0.1% hyaluronidase into the IVM medium and washing in with TLH/PVA. Denuded oocytes from each group were collected, stained for 10 min with TLH/PVA containing 10 mg/mL Hoechst-33342, and then examined under a fluorescence microscope (Nikon, Tokyo, Japan). To assess meiotic maturation, oocytes were classified into the following stages: germinal vesicle (GV), metaphase I (MI), anaphase/telophase I (A/TI), and MII. Four stages were evaluated based on the previously reported morphology of stained nuclei (35). Oocytes that underwent an initial polar body extraction were considered mature. The experiment was performed in triplicate.

### 2.7. Measurement of intracellular glutathione and reactive oxygen species levels

Mature oocytes were collected 42–44 h after IVM, and intracellular GSH and ROS levels were measured as previously described (36). In summary, intracellular GSH was detected in the ooplasm using CellTracker Blue 4-chloromethyl-6,8-difluoro-7-hydroxycoumarin (CMF_2_HC) (Invitrogen), and intracellular ROS levels were detected using the indicator 2′,7′-dichlorodihydrofluorescein diacetate (H_2_DCFDA) (Invitrogen). Twenty oocytes were placed in TLH/PVA supplemented with 10 μM CMF_2_HC dye or 10 μM H_2_DCFDA and then incubated for 30 min in the dark. After washing thrice with TLH/PVA, the dyed oocytes were placed into a 10 μL drop of TLH/PVA and then quantified using an epifluorescence microscope (TE300; Nikon) with an ultraviolet filter (370 nm for GSH; 460 nm for ROS). The fluorescence intensity was quantified using Image J software (NIH, Bethesda, MD, USA) and normalized to that of control oocytes. Three independent experiments were conducted (GSH samples, *n* = 60; ROS samples, *n* = 60 per group).

### 2.8. **Quantitative real-time polymerase chain reaction**

To analyze *CCL2* expression patterns in the three types of follicular cells before and after IVM, oocytes, CCs, and GCs were collected from aspirated pFF and sampled immediately. CCs and oocytes were sampled after 40–42 h of IVM in a medium containing pFF. GCs were cultured separately under the same conditions. CCs and oocytes were separated from the 60 COCs and isolated from each other by gentle pipetting with 0.1% hyaluronidase. All cells were washed twice in DPBS, placed in 1.5 mL microfuge tubes, and frozen at −80°C until analysis. qPCR was performed to analyze the expression of *CCL2* and 18 specific genes associated with particular functions, including the apoptosis-related genes: BCL2 associated X (*BAX*), BCL2 like 1 (*BCL2L1*), and caspase-3 (*CASP3*); the antioxidant-related genes: nuclear factor erythroid 2-related factor 2 (*NRF2*), superoxide dismutase 1 (*SOD1*), and superoxide dismutase 2 (*SOD2*); the cumulus expansion-/proliferation-related genes: pentraxin 3 (*PTX3*), TNF alpha induced protein 6 (*TNFAIP6*), CD44 molecule (Indian blood group) (*CD44*), and proliferating cell nuclear antigen (*PCNA*); the oocyte meiotic resumption-related gene: natriuretic peptide receptor 2 (*NPR2*); the developmental competence-related genes: zygote arrest 1 (*ZAR1*) and nucleophosmin/nucleoplasmin 2 (*NPM2*); and the CCL2 signaling pathway-related genes extracellular signal-regulated protein kinase 1 (*ERK1*), extracellular signal-regulated protein kinase 2 (*ERK2*), phosphoinositide-3-kinase regulatory subunit 1 (*PIK3R1*), AKT serine/threonine kinase 1 (*AKT1*), and nuclear factor kappa B subunit 1 (*NFKB1*). The mRNA expression levels of *CCL2* were analyzed in oocytes, CCs, and GCs before and after IVM. In CCs, we assessed the mRNA expression levels of *BAX, BCL2L1, CASP3, NRF2, SOD1, SOD2, PTX3, TNFAIP6, CD44, PCNA, NPR2, ERK1, ERK2, PIK3R1, AKT1*, and *NFKB1*, and in oocytes, we assessed the mRNA expression levels of *BAX, BCL2L1, CASP3, NRF2, SOD1, PCNA, ZAR1, NPM2, ERK1, ERK2, PIK3R1, AKT1*, and *NFKB1*. Supplementary Table 1 lists all primer sequences.

RNA extraction and cDNA synthesis were conducted as described in Section 2.4. qPCR was performed as previously described (37). The qPCR mixture for the CFX96 Touch RealTime PCR Detection System (Bio-Rad, Hercules, CA, USA), containing 1 μg synthesized cDNA, 2X SYBR Premix Ex Taq (TaKaRa Bio), and 10 pmol of specific primers (Macrogen). The reactions were run for 40 cycles of denaturation at 95°C for 15 s, annealing at 57°C for 15 s, and extension at 72°C for 24 s. Relative quantification was performed at a constant fluorescence intensity, using threshold cycle-based techniques. Relative mRNA expression (R) was determined using the following equation: *R* = 2 ^−[Δ*Ct sample*−Δ*Ct control*]^. *R*-values for glyceraldehyde 3-phosphate dehydrogenase (*GAPDH*) in CCs and GCs and *RN18S* in oocytes were used to normalize the R values for each gene. Statistical analyses were conducted using at least three independent replicates.

### 2.9. Western blotting

Protein expression and activation levels were measured by western blotting using previously described techniques (38). After IVM with CCL2 supplementation, CCs, and oocytes were separated using 0.1% hyaluronidase and sampled. All samples were stored at −80°C until analysis. Total protein was extracted from CCs and oocytes using a 100 X Halt™ protease and phosphatase inhibitor single-use cocktail and 0.5 M ethylenediaminetetraacetic acid solution (Thermo Fisher Scientific, Waltham, MA, USA). Using bovine serum albumin as a standard, the protein concentrations were assessed using a Pierce™ bicinchoninic acid protein assay kit (Thermo Fisher Scientific), according to the manufacturer's instructions. Lysates were resolved using 10% sodium dodecyl sulfate-polyacrylamide gel electrophoresis and electrophoretically transferred onto polyvinylidene fluoride membranes (Millipore, Billerica, MA, USA). After washing thrice for 10 min with Tris-buffered saline–Tween 20 buffer (0.2 μM Tris, 1.37 M NaCl, and 0.05% Tween 20), the membranes were blocked for 5 min at RT using EveryBlot blocking buffer (Bio-Rad) and then incubated with primary antibodies overnight at 4°C. The following primary antibodies were used rabbit anti-ERK1/2 (1:1,000; 9102S; Cell Signaling Technology), rabbit anti-phospho ERK1/2 (p-ERK1/2) (1:1,000; 9101S; Cell Signaling Technology), and rabbit anti-GAPDH (1:1,000; 2118S; Cell Signaling Technology). The following day, the membranes were washed thrice with Tris-buffered saline–Tween 20 buffer for 10 min on a shaker at 100 revolutions per min and then incubated with horseradish peroxidase-conjugated secondary antibodies (anti-rabbit) for 1.5 h at RT. A chemiluminescent substrate kit (4:1 mixture of SuperSignal West Pico PLUS chemiluminescent substrate and SuperSignal West Femto Maximum Sensitivity Substrate; Thermo Fisher Scientific) was used to visualize the target proteins. The protein bands were detected using a Lumino Graph II (ATTO Corporation), and the optical densities of the target proteins were determined using CS Analyzer 4 software. The expression levels of all proteins were normalized to that of GAPDH. All experiments were performed at least thrice.

### 2.10. Parthenogenetic activation and *in vitro* culture of porcine embryos

After IVM, CCs and oocytes were isolated from each other, as described above. MII oocytes from each group were selected and washed with a calcium-free TLH/PVA medium. PA was performed using an established procedure (39). The MII oocytes were washed twice with a 280 mM mannitol solution containing 0.01 mM CaCl_2_ and 0.05 mM MgCl_2_. The MII oocytes were loaded between the electrodes of the chamber in 2 mL of a 260 mM mannitol solution containing 0.001 mM CaCl_2_ and 0.05 mM MgCl_2_. An electrical pulsing machine (LF101; Nepa Gene, Chiba, Japan) was connected to the chamber, and the MII oocytes were stimulated by two 60s 120 V/mm direct -current pulses. After PA, the activated oocytes from each group were immediately transferred to a porcine zygote medium containing 5 μg/mL cytochalasin B, which served as an IVC culture medium (40). The oocytes were incubated at 39°C in a humidified atmosphere of 5% CO_2_ and 95% N_2_ for 4 h. After rinsing twice in a fresh IVC medium, the oocytes were placed into droplets of 25 μL fresh IVC medium (10 oocytes per droplet) covered with mineral oil. Embryos were cultured for 7 d at 39°C in a humidified incubator with 5% O_2_, 5% CO_2_, and 90% N_2_. The IVC culture medium was refreshed after 48 h (Day 2) and 96 h (Day 4) with 10% fetal bovine serum. The experiment was repeated thrice.

### 2.11. Evaluation of developmental competence and total cell count of blastocysts

The cleavage of PA embryos was assessed on Day 2. Normally cleaved embryos were classified into the 2–3 cell, 4–5 cell, and 6–8 cell embryo stages, excluding 1-cell embryos and fragmented embryos. Blastocyst formation was evaluated on Day 7, similar to a previous study (41). The blastocysts were divided into three groups based on their morphology: early, expanded, or hatched. To calculate the total cell number, on Day 7, blastocysts were rinsed in TLH/PVA, and fixed in 4% paraformaldehyde in PBS/PVA and then stained for 10 min with 5 g/mL Hoechst-33342. Blastocysts fixed in 100% glycerol droplets were mounted on glass slides and counted manually using a fluorescence microscope (TE300, Nikon) with an ultraviolet filter (370 nm). The experiment was repeated thrice.

### 2.12. Statistical analysis

All experiments in this study were conducted at least thrice. For each replicate, ovaries obtained on the same day from the same slaughterhouse were used. Data were statistically analyzed using GraphPad Prism (GraphPad Software, San Diego, CA, USA) and SPSS version 12.0 (SPSS, IBM, Armonk, NY, USA). Following a one-way analysis of variance (ANOVA) test, Duncan's multiple range test was used to examine percentage data (rates of nuclear maturation and embryonic development) and average data (ELISA data, intracellular GSH and ROS levels in oocytes, and the total number of cells in blastocysts). An unpaired two-tailed Student's *t*-test was used to examine average data (Western blotting and qPCR of follicular cells before and after IVM) between two groups. Data are presented as the mean ± standard error of the mean. Differences were considered statistically significant at *p* < 0.05.

## 3. Results

### 3.1. Measurement of CCL2 concentration in pFF

ELISA was performed to determine the concentration of CCL2 in follicular fluids produced by porcine follicles of three different sizes (small, medium, and large). The maturation medium, M199 was used as a negative control. CCL2 was detected in pFF from follicles of all sizes. The CCL2 concentration in pFF increased in proportion to follicle size. The CCL2 concentration in the large group (39.0 ± 0.3 pg/mL) was significantly higher than that in the small group (13.7 ± 0.9 pg/mL) and the medium group (24.2 ± 2.6 pg/mL). The CCL2 concentrations determined using a standard curve are shown in Table 1.

**Table 1:** Concentration of CCL2 in pFF from different follicle size (small, medium, and large).

	**Size of follicles**			**M199**
	**Small (1–2 mm)**	**Medium (3–7 mm)**	**Large (**≥**8 mm)**	
CCL2 (pg/mL)	13.7 ± 0.9^a^	24.2 ± 2.6^b^	39.0 ± 0.3^c^	ND

The data shown represents mean ± SEM from four independent replicates.

^a, b, c^Values with different superscripts within a row differ significantly (p < 0.05).

ND, not detected.

### 3.2. Identification of *CCL2* in porcine follicular cells

**Figure 1:**
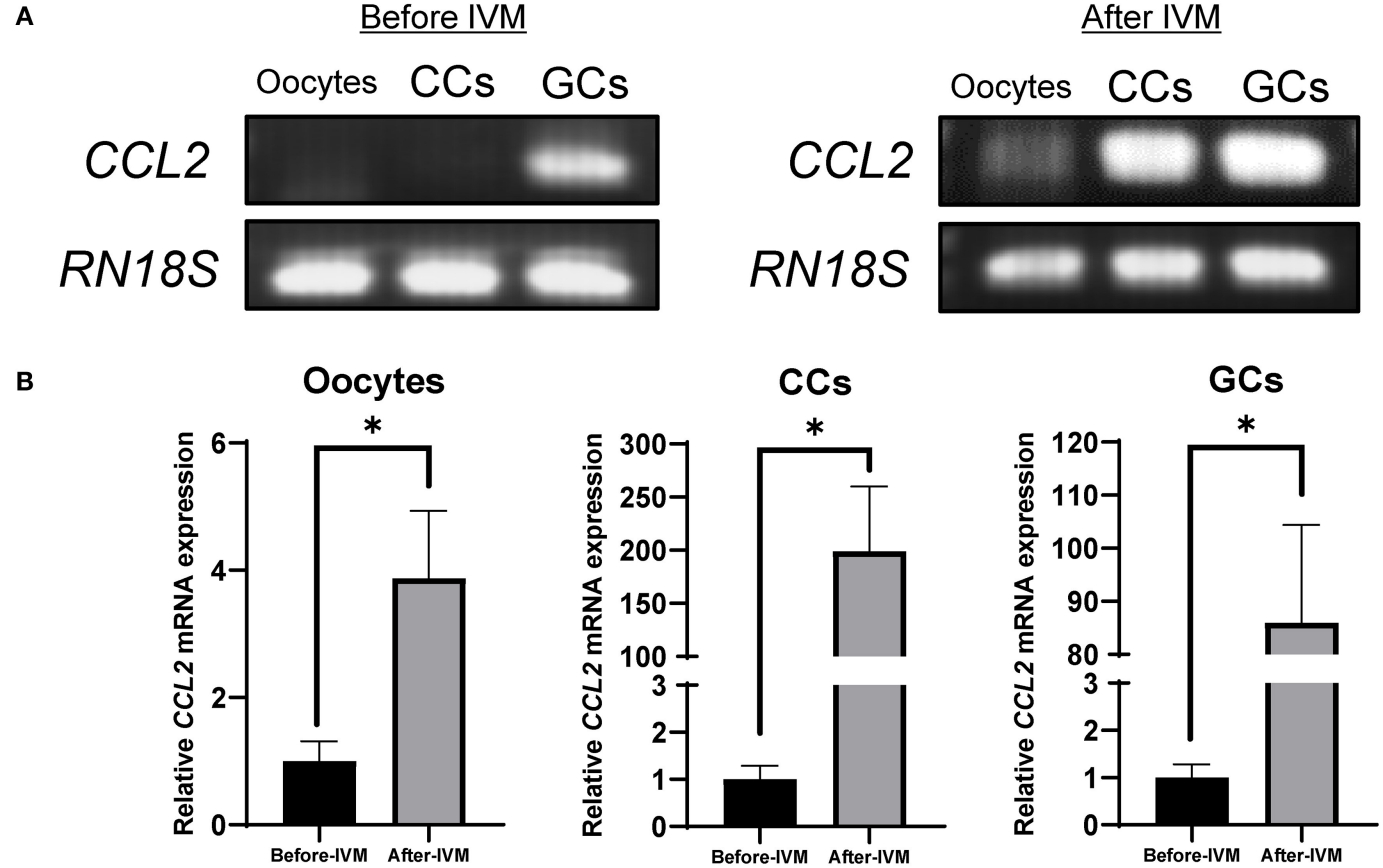
Expression of *CCL2* in porcine oocytes, CCs, and GCs was determined by RT-PCR **(A)** and qPCR **(B)**. *CCL2* mRNA levels were detected in porcine follicular cells, before and after IVM. *RN18S* served as a ubiquitously expressed gene control. The experiment was conducted three times. *p < 0.05.

RT-PCR and qPCR were performed to analyze the expression of *CCL2* in porcine follicular cells (oocytes, CCs, and GCs), before and after IVM. *CCL2* was expressed in GCs, before and after IVM. In contrast, in oocytes and CCs, *CCL2* was expressed only in the IVM group. After IVM, *CCL2* was weakly expressed in oocytes (Figure 1A). The mRNA levels of *CCL2* were quantified using qPCR. As shown in Figure 1B, these levels increased significantly in all three porcine follicular cell types after IVM.

### 3.3. Localization of CCL2 and CCR2 in follicular cells before and after IVM

**Figure 2:**
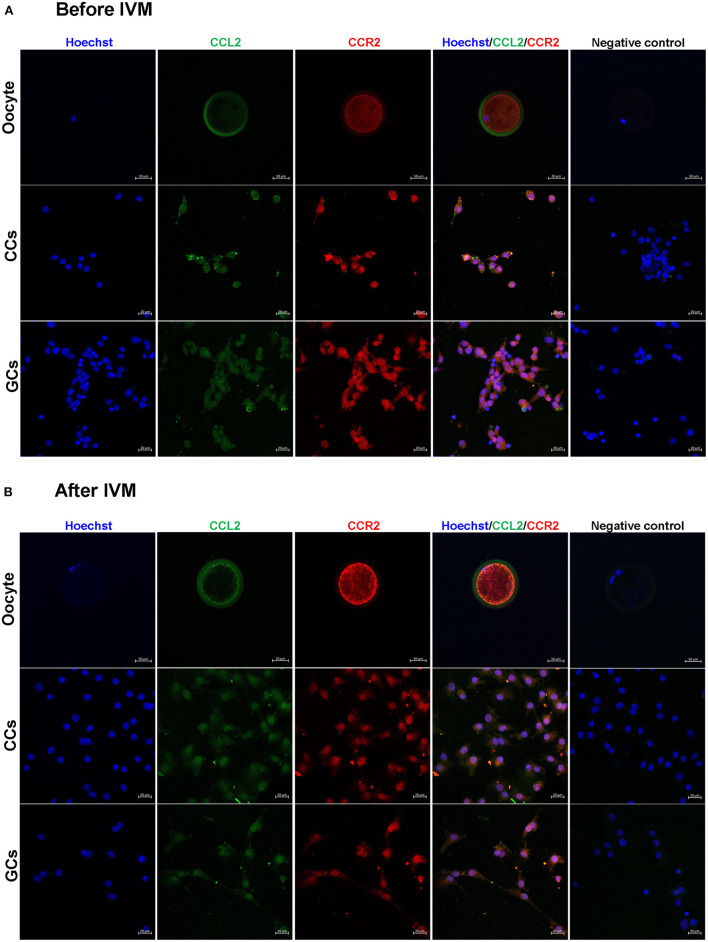
Detection and localization of CCL2 and its receptor, CCR2, in porcine follicular cells before **(A)** and after **(B)** IVM. Blue = Hoechst, green = CCL2, red = CCR2. No staining of CCL2 and CCR2 was observed in the negative control. CCL2 and CCR2 immunostaining was observed in all porcine follicular cells before and after IVM. Scale bar = 20 μm in oocyte and 50 μm in CCs and GCs.

The localization of CCL2 and its receptor, CCR2 in oocytes, CCs, and GCs were determined before and after IVM. The expression of CCL2 and CCR2 was observed in all cell types. In CCs and GCs, CCL2 and CCR2 were expressed in both the nucleus and cytoplasm, before and after IVM, and no significant difference was observed after IVM. Interestingly, in oocytes, CCL2 was strongly expressed in the zona pellucida before IVM (Figure 2A), but after IVM, it was strongly expressed in the zona pellucida and at the edge of the oocyte cytoplasm (Figure 2B). CCR2 exhibited high levels of overall expression in the oocyte cytoplasm both before and after IVM.

### 3.4. Effect of CCL2 supplementation during IVM on oocyte nuclear maturation

To investigate the effect of CCL2 treatment on the nuclear maturation of porcine oocytes during IVM, mature oocytes were evaluated at four stages: GV, MI, A/TI, and MII. After 42 h of IVM, the group supplemented with 100 ng/mL CCL2 exhibited a significantly (*p* < 0.05) higher M II rate (91.6 ± 3.6%) than the control group (81.8 ± 1.8%). In contrast, no significant differences were found between the other CCL2-treated groups (1 ng/mL: 87.6 ± 2.1%; 10 ng/mL: 91.3 ± 3.4%) and the control group (Table 2).

**Table 2:** Effect of CCL2 supplementation during IVM on nuclear maturation.

**CCL2 concentration (ng/mL)**	No. of oocytes cultured for maturation^*^	**Mean** ±**SEM (%) oocytes at the stage of**			
		**Germinal vesicle**	**Metaphase I**	**Anaphase I and Telophase I**	**Metaphase II**
0 (Control)	158	1.8 ± 1.0	14.0 ± 1.8	2.3 ± 1.5	81.8 ± 1.8^a^
1	158	1.4 ± 1.4	9.2 ± 3.7	1.2 ± 1.2	87.6 ± 2.1^a, b^
10	166	0.0 ± 0.0	5.2 ± 3.7	2.8 ± 2.8	91.3 ± 3.4^a, b^
100	158	0.0 ± 0.0	5.8 ± 3.9	1.2 ± 0.6	91.7 ± 3.6^b^

^*^Three times replicates.

^a, b^Values with different superscripts within a column differ significantly (p < 0.05).

### 3.5. Effect of CCL2 supplementation during IVM on oocyte intracellular GSH and ROS levels

**Figure 3:**
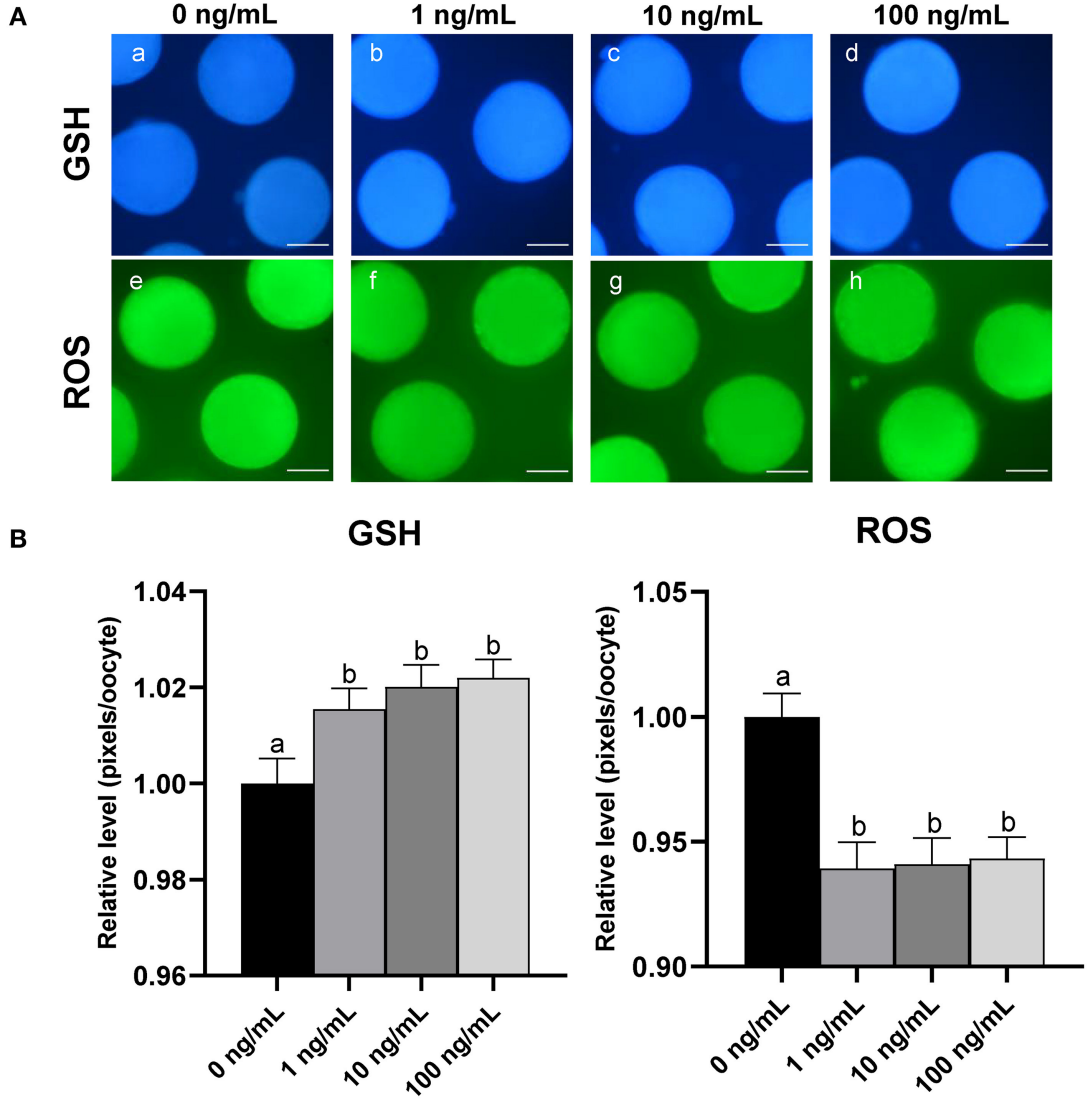
Effect of CCL2 supplementation during IVM on cytoplasmic maturation. **(A)** Oocytes were stained with Cell Tracker Blue (a–d) and H_2_DCFDA (e–h) to detect intracellular levels of GSH and ROS, respectively. MII oocytes were derived from the control IVM system and the IVM system supplemented with various concentrations of CCL2. **(B)** Relative levels of intracellular GSH and ROS in *in vitro* mature porcine oocytes treated with CCL2 during IVM. Bars with different letters (a, b) are significantly different (*p* < 0.05). The experiment was replicated three times. Scale bar = 50 μm.

To investigate the effect of CCL2 treatment on oocyte cytoplasmic maturation during IVM, M II stage oocytes from each group were stained to evaluate intracellular GSH and ROS levels. Intracellular GSH levels were significantly (*p* < 0.05) increased in all CCL2-treated groups, compared to the control group. Intracellular ROS levels were significantly (*p* < 0.05) decreased in all CCL2-treated groups (Figures 3A, B). Therefore, CCL2 supplementation during IVM significantly affected the intracellular GSH and ROS levels in mature oocytes.

### 3.6. Effect of CCL2 supplementation during IVM on the expression levels of various genes in CCs and oocytes

To investigate the effects of CCL2 treatment on porcine COCs during IVM, qPCR was used to analyze the expression of various genes in CCs and oocytes. To examine the effect of CCL2 on the expression of apoptosis-, antioxidant-, cumulus expansion-, and proliferation-related genes, the mRNA expression levels of *BAX, BCL2L1, CASP3, NRF2, SOD1, SOD2, PTX3, TNFAIP6, CD44*, and *PCNA* were assessed in CCs and oocytes. The mRNA expression of *NPR2*, a gene related to the resumption of meiosis in oocytes, was confirmed in CCs, and that of *ZAR1* and *NPM2*, which are maternal-effect genes, was confirmed in oocytes.

Among apoptosis-related genes, the pro-apoptotic gene *BAX* exhibited a significantly (*p* < 0.05) decreased level of mRNA expression in CCs and oocytes in all CCL2-treated groups. *CASP3* levels were significantly (*p* < 0.05) decreased in CCs in the 100 ng/mL treatment group, compared. *CASP3* levels were also, significantly (*p* < 0.05) decreased in oocytes in all treatment groups, compared to control. Among antioxidant-related genes, the levels of *SOD1* and *SOD2* were significantly (*p* < 0.05) increased in CCs in the 100 ng/mL group, compared to the control group. The level of *NRF2* was significantly (*p* < 0.05) increased in oocytes in the 10 ng/mL group. Among cumulus expansion- and proliferation-related genes, *PTX3* levels were significantly (*p* < 0.05) increased in CCs in the 10 ng/mL group, compared to the control. *CD44* levels were significantly (*p* < 0.05) increased in CCs in all treatment groups. *PCNA* levels were not significantly different in any of the treatment groups compared to those in the control. However, *PCNA* levels in oocytes were significantly (*p* < 0.05) increased in the 10 ng/mL group compared to those in the 100 ng/mL group.

**Figure 4:**
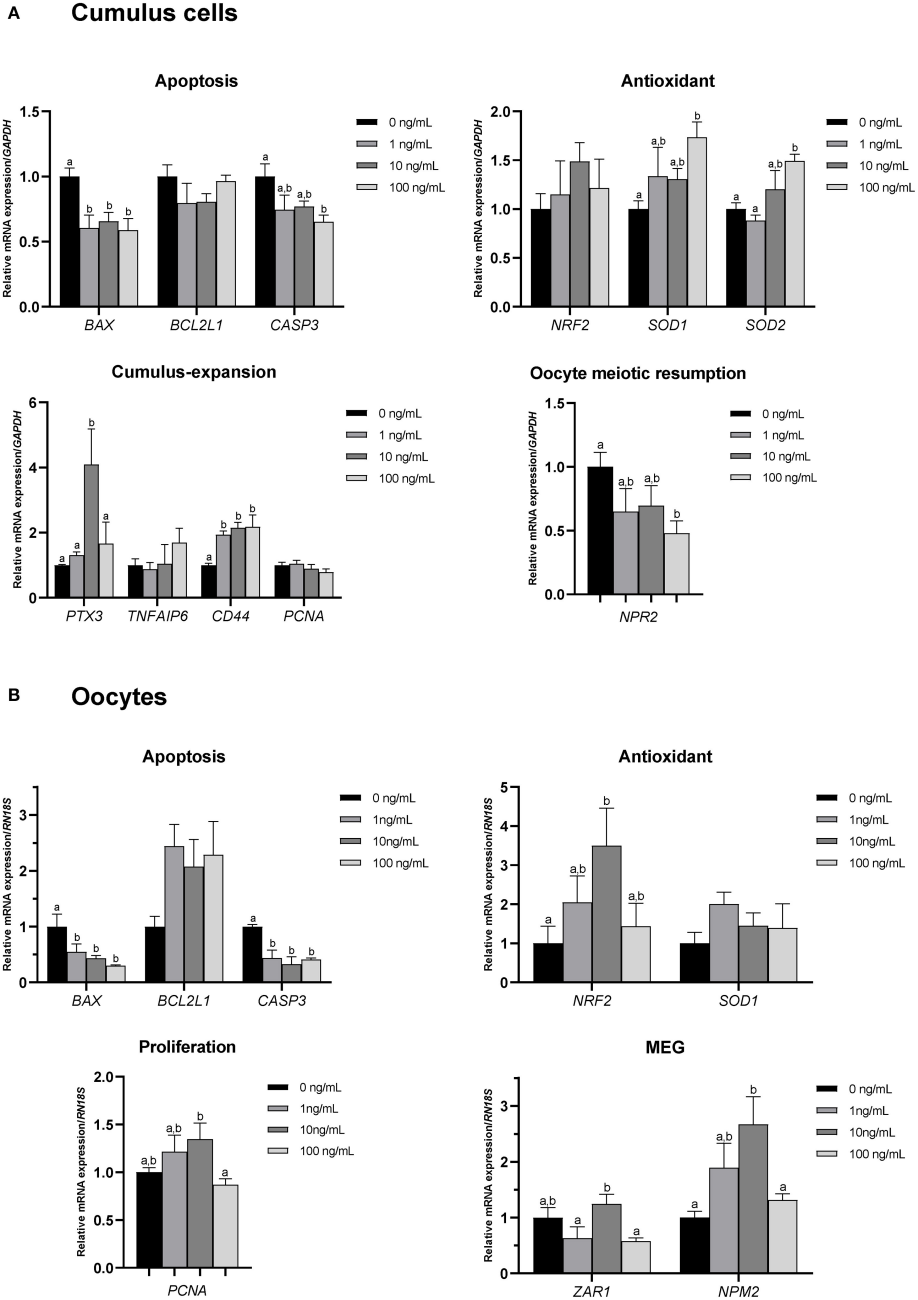
mRNA levels of apoptosis-, antioxidant-, cumulus expansion/proliferation-, and oocyte meiotic resumption-related genes and maternal-effect genes (MEGs). Levels of *BAX, BCL2L1, CASP3, NRF2, SOD1, SOD2, PTX3, TNFAIP6, CD44, PCNA*, and *NPR2* were assessed in groups of CCs **(A)**, and levels of *BAX, BCL2L1, CASP3, NRF2, SOD1, PCNA, ZAR1*, and *NPM2* were assessed in groups of oocytes **(B)** supplemented with 0, 1, 10, and 100 ng/mL of CCL2 during IVM. Data is represented as mean ± SEM. The experiment was replicated at least three times. Bars with different letters (a, b) are significantly different (*p* < 0.05).

The level of *NPR2*, a gene associated with oocyte meiotic resumption, was significantly (*p* < 0.05) decreased in CCs in the 100 ng/mL CCL2 treatment group, compared to the control. Levels of the maternal-effect gene, *ZAR1* in oocytes exhibited no significant differences between any treatment groups and the control. In contrast, the expression of the maternal-effect gene *NPM2* was significantly increased in oocytes treated with 10 ng/mL CCL2 (Figures 4A, B).

### 3.7. Effect of CCL2 supplementation during IVM on CCL2 signaling pathway-related gene expression levels in CCs and oocytes

qPCR was used to assess the mRNA expression of several CCL2 signaling pathway-related genes, including *ERK1, ERK2, PIK3R1, AKT1*, and *NFKB1*, in CCs and oocytes after IVM.

**Figure 5:**
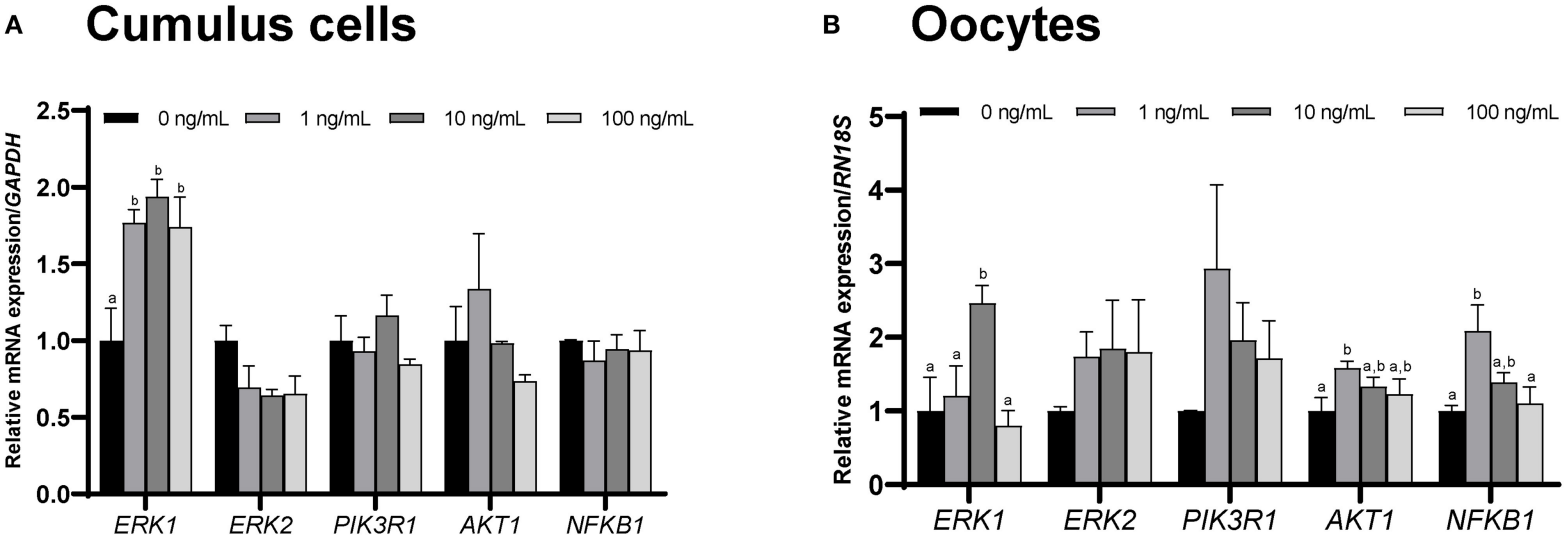
mRNA levels of CCL2 signaling pathway-related genes. Levels of *ERK1, ERK2, PIK3R1, AKT1*, and *NFKB1* were assessed in groups of CCs **(A)** and oocytes **(B)** supplemented with 0, 1, 10, and 100 ng/mL of CCL2 during IVM. Data is represented as mean ± SEM. The experiment was replicated three times. Bars with different letters (a, b) are significantly different (*p* < 0.05).

The level of *ERK1* in CCs and oocytes was significantly (*p* < 0.05) increased in the 10 ng/mL CCL2 treatment group, compared to that in the control. The levels of *AKT1* and *NFKB1* in oocytes were significantly (*p* < 0.05) increased in the 1 ng/mL treatment group, compared to those in the control (Figures 5A, B).

### 3.8. Effect of CCL2 supplementation on ERK1/2 protein expression levels in CCs and oocytes during IVM

The mRNA expression of *ERK1* was significantly increased in CCs and oocytes in the 10 ng/mL CCL2-treated group, compared to that in the control. Therefore, the protein expression of ERK1/2 was also analyzed.

**Figure 6:**
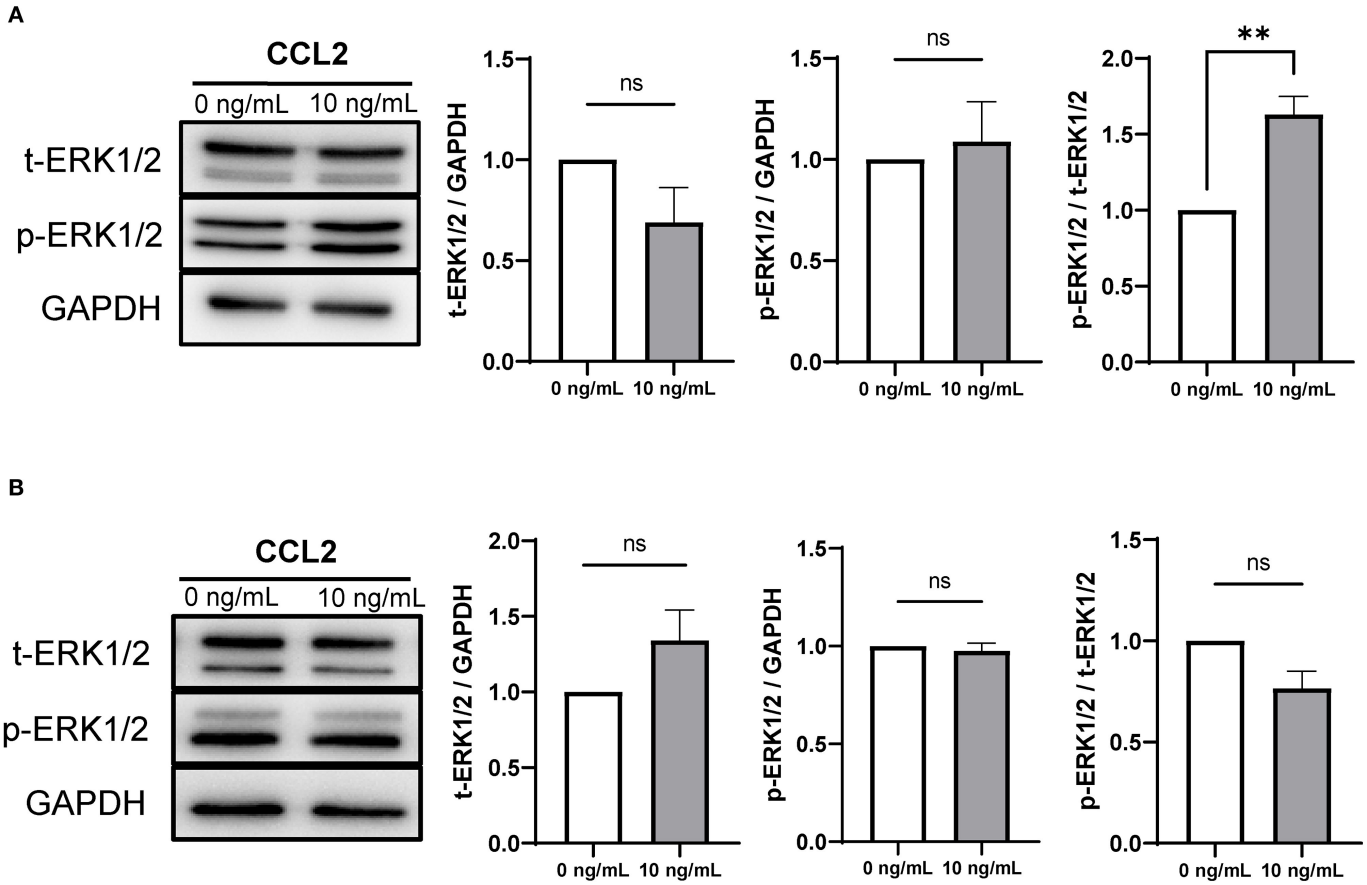
Expression and phosphorylation levels of ERK1/2 in porcine CCs and oocytes after IVM. Western blots of t-ERK1/2, p-ERK1/2, and GAPDH in CCs **(A)** and oocytes **(B)**. Levels of t-ERK1/ 2 and p-ERK1/2 and the ratio of p-ERK1/2 to t-ERK1/2 in CCs and oocytes are shown. The results were normalized to those of GAPDH. Data is represented as mean ± SEM. The experiment was replicated three times. **p < 0.01; ns, not significant (*p* > 0.05).

In CCs, the ratio of phosphorylated ERK1/2 (p-ERK1/2) to total ERK1/2 (t-ERK1/2) was significantly (*p* < 0.01) increased only in the 10 ng/mL treatment group, compared to that in the control (Figure 6A). In contrast, in oocytes, there was no significant difference between the protein expression ratios of t-ERK1/2 to GAPDH, p-ERK1/2 to GAPDH, and p-ERK1/2 to t-ERK1/2 (Figure 6B).

### 3.9. Effect of CCL2 supplementation during IVM on embryonic development after PA

**Figure 7:**
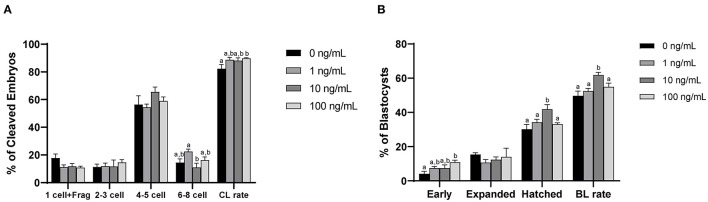
Effect of CCL2 supplementation during IVM on the cleavage (CL) pattern **(A)** and blastocyst (BL) formation pattern **(B)** of PA embryos. Within each end point, bars with different letters (a, b) differ significantly (*p* < 0.05) for different concentrations of CCL2. Fragmentation; Frag. The cleavage and blastocyst rates were evaluated on days 2 and 7 after PA, respectively. The experiment was replicated three times.

After PA, the cleavage rate was significantly higher (*p* < 0.05) in the 100 ng/mL CCL2-treated group than in the control group (*p* < 0.05; Figure 7A). In contrast, the blastocysts formation rate was significantly higher in the 10 ng/mL CCL2-treated group than in the control group (*p* < 0.05; Figure 7B). However, the patterns of these two results were inconsistent. The rate of early blastocysts among PA embryos was significantly increased in the 100 ng/mL CCL2-treated group (*p* < 0.05), whereas the rate of hatched blastocysts in the 10 ng/mL CCL2-treated group was significantly higher than that in the control and other CCL2-treated groups (*p* < 0.05). However, the total cell number in blastocysts was not significantly different than in the control (Table 3).

**Table 3:** Effect of CCL2 supplementation during IVM on embryonic developmental potential after PA.

**CCL2 concentration (ng/mL)**	No. of Embryos cultured, N^*^	**No. (%) of Embryos developed to**		Total cell number in blastocysts (n^**^)
		≥**2-cell**	**Blastocyst**	
0 (Control)	148	122 (82.3 ± 3.0)^a^	74 (49.8 ± 2.7)^a^	59.3 ± 4.2 (74)
1	160	142 (88.8 ± 1.6)^a, b^	84 (52.5 ± 1.6)^a^	62.3 ± 4.1 (84)
10	162	143 (88.2 ± 2.0)^a, b^	100 (61.8 ± 1.7)^b^	69.7 ± 1.4 (100)
100	166	149 (89.8 ± 0.5)^b^	91 (54.9 ± 2.2)^a^	50.5 ± 9.7 (91)

N^*^: Three times replicates. n^**^: Number of blastocysts examined.

^a, b^Values with different superscripts within a column differ significantly (p < 0.05).

## 4. Discussion

Ovulation is a complex physiological process in which ovulatory follicles release mature oocytes. It is considered as an inflammatory response where follicular tissue is ruptured and repaired due to the inflammatory mediators (42, 43). GCs function as immune cells during ovulation. They are the source of CCL2, which is responsible for recruiting macrophages (10, 44). Numerous cytokines in human follicular fluid influence follicle development and maturation, ovulation, and corpus luteum formation (45). Follicular fluid with a high CCL2 concentration contains mature oocytes (46). The expression levels of *CCL2* mRNA in human ovarian stroma increase toward the late ovulatory phase and decrease again in the postovulatory phase (47). As the size of a follicle increases, approaching ovulation, the follicular concentration of CCL2 also increases. Similarly, the RNA expression of CCL2 in bovine GCs and theca cells increases with the size of the follicle (25). Following these reports, the present study showed that CCL2 was present in pFF and that its concentration increased significantly with the size of the ovulatory follicle. During follicular development, increasing concentration of CCL2 may be involved in ovulation.

In IVM, hormones, such as eCG and hCG, are added to the culture medium. Similar to the luteinizing hormone (LH), these hormones equip oocytes with the developmental capacity for maturation, *in vivo* (48). The LH surge induces CCL2 expression in ovarian stromal and granulosa-lutein cells, and promotes follicle growth, which results in an influx of monocytes into the preovulatory follicle (45). The concentration of CCL2 in follicular fluid samples significantly increases after hCG administration (49). Hormones play a major role in increasing the mRNA expression of *CCL2*. In this study, for the first time, we observed the localization of CCL2 and CCR2 in porcine follicular cells *via* immunostaining. CCL2 and CCR2 expression have also been observed in feline COCs *via* immunostaining (28). CCL2-CCR2 signaling initiates G protein-coupled receptor signaling in leukocytes, which includes the activation of PI3K/Akt/mTOR, a critical pathway also associated with follicular activation and survival (50, 51). CCR2 plays a crucial role in the regulation of murine folliculogenesis (52). Antral follicle development and oocyte growth and maturation occur together; moreover, antral folliculogenesis affects oocyte development and maturation (53). Therefore, CCL2 and CCR2, present in porcine follicular cells, are involved in follicle growth and oocyte maturation.

Successful IVM requires both nuclear and cytoplasmic maturation of oocytes (54). The GC ligand natriuretic peptide type C (NPPC) and its receptor, NPR2, are important for maintaining meiotic arrest in CCs. However, when the LH surge occurs, *NPPC* and *NPR2* mRNA expression is decreased, leading to the resumption of oocyte meiosis (55, 56). We observed that in the 100 ng/mL CCL2 treatment group, the proportion of MII stage oocytes increased after IVM, whereas the mRNA levels of NPR2 decreased in CCs. Macrophage colony-stimulating factor, a similar cytokine to CCL2 with the same role in ovarian physiology, decreases *NPR2* mRNA expression and increases the rate of germinal vesicle breakdown in oocytes (57, 58). GSH and ROS levels in oocytes are important indicators of cytoplasmic maturation after IVM (59). GSH levels increased and ROS levels decreased in oocytes treated with CCL2 at all concentrations. The mRNA levels of *NRF2* in oocytes increased in the 10 ng/mL CCL2-treated group. The Nrf2-antioxidant response element signaling pathway is a major defense mechanism against oxidative stress. GSH protects cells against oxidative stress by attenuating ROS levels and increasing NRF2 expression in the brain (60). CCL2 supplementation during IVM promotes both nuclear and cytoplasmic maturation of oocytes.

The mRNA expression levels of *BAX* and *CASP3* decreased in both CCs and oocytes in the 100 ng/mL CCL2 treatment group. When the expression of these pro-apoptotic genes is inhibited or defective, dopamine-induced apoptosis is prevented in mice (61). As previously reported, CCL2 and FBN-1 are regulated by BMP15 which prevents apoptosis in porcine CCs (26). CCL2 treatment during cardiac myocyte culture partially inhibits BAX induction and, inhibited caspase 3-like activation, protecting them from hypoxia-induced apoptosis (62). Our results indicate that CCL2 downregulates *BAX* and *CASP3* in CCs and oocytes during IVM, which may inhibit apoptosis. Among antioxidant-related genes, *SOD1* and *SOD2* exhibited increased mRNA expression levels in CCs treated with 100 ng/mL CCL2. Although the association between CCL2 and these two antioxidant-related genes is unknown, C-C motif chemokine ligand 5, a member of the same chemokine family, has been shown to enhance glutathione peroxidase 1 antioxidant activity in mice with mild traumatic brain injury (63). Therefore, CCL2 supplementation during IVM may reduce apoptosis by upregulating antioxidant-related genes in CC and oocytes.

Of the CCL2 signaling pathway-related genes, *ERK1* exhibited increased mRNA levels in CCs and oocytes treated with 10 ng/mL CCL2, and this trend was confirmed even at the protein level. The protein expression ratio of phosphorylated ERK1/2 (p-ERK1/2) to t-ERK1/2 was increased only in CCs treated with 10 ng/mL of CCL2. Implying that along with the LH surge, ERK1/2 activation is required for cumulus expansion and the resumption of meiosis in oocytes (64). The mRNA expression levels of *PTX3* and *CD44* were also increased in CCs. Particularly, PTX3 was associated with ERK1/2 activation. In pigs, ERK1/2 phosphorylation is a key factor in mitogen-activated protein kinase (MAPK) signaling in CCs and is induced by the exogenous ganglioside GT1b. It is shown that ERK1/2 phosphorylation enhances oocyte meiotic maturation and the expression of cumulus expansion factors including HAS2, TNFAIP6, and PTX3 (65). Similar to our results, a previous study showed that the mRNA expression of *PTX3* increased in the 10 ng/mL CCL2 treatment group (28). CD44 is another crucial molecule involved in cumulus expansion during porcine oocyte maturation (66). Similar to its functions in CCs shown here, CCL2 induces the activation of the MAPKs-ERK, c-Jun N-terminal kinase and p38 in human endothelial cells (67). In the MAPK pathway, CCL2 strongly activates p-ERK1/2 in porcine luminal epithelial cells (68). CCL2 supplementation during IVM eventually aids in cumulus-expansion. However, further studies are needed to determine whether CCL2 affects ERK1/2 activation in oocytes and identify the signaling pathway involved.

After PA, the cleavage rate increased in the 100 ng/mL CCL2 treatment group. The blastocysts formation rate increased in the 10 ng/mL CCL2 treatment group; in particular, the rate of blastocyst formation during the hatching stage was increased. Previous mammalian studies have reported increased GSH levels and decreased ROS levels in oocytes during IVM, which have been associated with improved developmental competence (69, 70). In the 10 ng/mL CCL2 treatment group, the mRNA expression levels of *NPM2*, a maternal-effect gene, were also increased. Maternal-effect genes accumulate maternal factors that regulate oocyte differentiation during oogenesis and enable early embryo development and the initial establishment of embryonic cell lineages. NPM2 which is expressed in the ooplasm after germinal vesicle breakdown, is essential for the transition from the zygote to the two-cell stage during subsequent embryonic development (71, 72). CCL2 treatment during IVM enhances embryonic development after PA by upregulating maternal-effect genes in porcine oocytes. Altogether, our findings clarify the effect of CCL2 supplementation during porcine IVM on oocyte maturation and subsequent embryo development, and this study may inform the discovery of new substances that can be used to supplement IVM culture medium.

## 5. Conclusion

In conclusion, CCL2 was present in pFF and all the three types of follicular cells. CCL2 supplementation during the IVM of porcine COCs promoted both nuclear and cytoplasmic maturation by downregulating pro-apoptotic genes and upregulating cumulus expansion- and antioxidant-related genes. This study demonstrated for the first time that an increased concentration of CCL2 in pFF during IVM, along with increased *CCL2* expression in follicular cells, enhanced porcine oocyte maturation and embryonic development following PA. Taken together, the results showed an overall beneficial effect upon IVM in the 10 ng/mL CCL2 treatment group. Our findings primarily provide novel insight into the effect of CCL2 on the maturation of porcine oocytes and may contribute to the enhancement of porcine IVM systems and associated technologies.

## Data availability statement

The datasets presented in this study can be found in online repositories. The names of the repository/repositories and accession number(s) can be found in the article/Supplementary material.

## Author contributions

SK, EK, and S-HH: conceptualization, validation, writing-original draft preparation, and writing-review and editing. SK, DO, HC, MK, LC, and JL: methodology and formal analysis. SK, DO, HC, MK, LC, JL, AJ, and ZH: investigation. S-HH: funding acquisition. All authors have read and agreed to the published version of the manuscript.
