# Supplementary material for: The effect of C–C motif chemokine ligand 2 supplementation on in vitro maturation of porcine cumulus-oocyte complexes and subsequent developmental competence after parthenogenetic activation
Source: Front Vet Sci. 2023 Mar 13;10:1136705. doi: 10.3389/fvets.2023.1136705 (PMC10040565; doi:10.3389/fvets.2023.1136705)
Supplement: Supplementary file 1 [file Table_1.docx]

Supplementary Material

# Supplementary Table 1

# Primer sequences used for qRT-PCR.

| **mRNA** | **Primer sequences** | **Tm (℃)** | **Product**  **size (bp)** | **GenBank** |
| --- | --- | --- | --- | --- |
|  |  |  |  | **accession number** |
| *RN18S* | F: 5’-CGCGGTTCTATTTTGTTGGT-3’ | 57 | 219 | NR_046261.1 |
|  | R: 5’-AGTCGGCATCGTTTATGGTC-3’ | 58 |  |  |
| *GAPDH* | F: 5’-GTCGGTTGTGGATCTGACCT-3’ | 60 | 374 | NC_010447.5 |
|  | R: 5’-TTGACGAAGTGGTCGTTGAG-3’ | 58 |  |  |
| *CCL2* | F: 5’-ATTAATTCTCCAGTCACCTGCT-3’ | 58 | 152 | NM_214214.1 |
|  | R: 5’-GGTTCTGCACAGATCTCCTT-3’ | 58 |  |  |
| *BAX* | F: 5’-TGCCTCAGGATGCATCTACC-3’ | 61 | 199 | XM_003127290 |
|  | R: 5’-AAGTAGAAAAGCGCGACCAC-3’ | 58 |  |  |
| *BCL2L1* | F: 5’-AATGACCACCTAGAGCCTTG-3’ | 58 | 182 | NM_214285 |
|  | R: 5’-GGTCATTTCCGACTGAAGAG-3’ | 58 |  |  |
| *CASP3* | F: 5’-CGTGCTTCTAAGCCATGGTG-3’ | 58 | 186 | NM_214131.1 |
|  | R: 5’-GTCCCACTGTCCGTCTCAAT-3’ | 58 |  |  |
| *NRF2* | F: 5’-CCCATTCACAAAAGACAAACATTC-3’ | 58 | 72 | XM_021075133.1 |
|  | R: 5’-GCTTTTGCCCTTAGCTCATCTC-3’ | 60 |  |  |
| *SOD1* | F: 5’-GTGCAGGGCACCATCTACTT-3’ | 60 | 222 | NM_001190422.1 |
|  | R: 5’- AGTCACATTGCCCAGGTCTC-3’ | 60 |  |  |
| *SOD2* | F: 5’-GACAAATCTGAGCCCTAACG-3’ | 61 | 191 | NM_214127.2 |
|  | R: 5’-GTTAGAACAAGCGGCAATCT-3’ | 60 |  |  |
| *PTX3* | F: 5’-AGACTTTATGCCATGGTGCT-3’ | 55 | 195 | NM_001244783.1 |
|  | R: 5’-TGACAGTGAGCAATGAACAA-3’ | 53 |  |  |
| *TNFAIP6* | F: 5’-TCATAACTCCATATGGCTTGAAC-3’ | 57 | 396 | NM_001159607.1 |
|  | R: 5’-TCTTCGTACTCATTTGGGAAGCC-3’ | 58 |  |  |
| *CD44* | F: 5’ -AGTCAAGAAGGTGAGGCAAA-3’ | 56 | 175 | XM_021085286.1 |
|  | R: 5’ –TGCCATTGTTAATCACCAGC-3’ | 56 |  |  |
| *PCNA* | F: 5’-CCTGTGCAAAAGATGGAGTG-3’ | 58 | 187 | NM_001291925.1 |
|  | R: 5’-GGAGAGAGTGGAGTGGCTTTT-3’ | 61 |  |  |
| *NPR2* | F: 5’- GGCACAGGAATCACCTTCAT-3’ | 60 | 364 | AY550069 |
|  | R: 5’-TGAAGCGAGTGAGATGGTTG-3’ | 60 |  |  |
| *ZAR1* | F: 5’-CTCCTGCCCAGTAAAACTTC-3’ | 56 | 196 | NM_001129956.1 |
|  | R: 5’-AAAAAGGCTCACTTGTCTGC-3’ | 57 |  |  |
| *NPM2* | F: 5’-GCTCTGGACCTGTGTTCCTC-3’ | 60 | 220 | NM_001195362.1 |
|  | R: 5’-GCTGCACTTGTCTGCTTCTG-3’ | 60 |  |  |
| *ERK1* | F: 5’-ATCACAGTGGAGGAAGCACT-3’ | 58 | 202 | XM_021088019 |
|  | R: 5’-GAGGCATCTGTCCAGGTTAG-3’ | 61 |  |  |
| *ERK2* | F: 5’-AGTCCATCGACATCTGGTCT-3’ | 58 | 240 | XM_021088019 |
|  | R: 5’-GAGCTTTGGAGTCAGCATTT-3’ | 56 |  |  |
| *PI3KR1* | F: 5’-CCACTACCGGAATGAATCTC-3’ | 55 | 211 | XM_021076847.1 |
|  | R: 5’-TTCCTGGGAAGTACGGGTAT-3’ | 57 |  |  |
| *AKT1* | F: 5’-CCACTACCGGAATGAATCTC-3’ | 58 | 208 | NM_001159776.1 |
|  | R: 5’-TTCCTGGGAAGTACGGGTAT-3’ | 57 |  |  |
| *NFKB1* | F: 5’-CTACCAGACACCCTTGCACT-3’ | 58 | 222 | NM_001048232 |
|  | R: 5’-ATAGCGTTCAGACCTTCACC-3’ | 58 |  |  |

F: Forward, R: Reverse, Tm (℃): Melting temperature
